# The Effectiveness of Psycho-Educational and Cognitive-Behavioral Counseling on Female Sexual Dysfunction

**DOI:** 10.1055/s-0040-1712483

**Published:** 2020-06

**Authors:** Firoozeh Mirzaee, Atefeh Ahmadi, Zahra Zangiabadi, Moghaddameh Mirzaee

**Affiliations:** 1 Razi Nursing and Midwifery Faculty, Kerman University of Medical Sciences, Kerman, Iran; 2 Department of Epidemiology and Biostatistics, School of Public Health, Kerman University of Medical Sciences, Kerman, Iran

**Keywords:** cognitive-behavioral counseling, psychoeducational counseling, female sexual function index

## Abstract

**Introduction** Sexual function is a multidimensional phenomenon that is affected by many biological and psychological factors. Cognitive-behavioral sex therapies are among the most common nonpharmacological approaches to psychosexual problems. The purpose of the present study was to investigate the effectiveness of psychoeducational and cognitive-behavioral counseling on female sexual dysfunction.

**Methods** The present study was a clinical trial with intervention and control groups. The study population consisted of women referring to the general clinic of a governmental hospital in Iran. After completing the demographic questionnaire and Female Sexual Function Index (FSFI), those who obtained the cutoff score ≤ 28 were contacted and invited to participate in the study. Convenience sampling method was used and 35 subjects were randomly allocated for each group. Eight counseling sessions were held for the intervention group (two/week/1.5 hour). Post-test was taken from both groups after 1 month, and the results were statistically analyzed by PASW Statistics for Windows, Version 18 (SPSS Inc., Chicago, IL, USA).

**Results** The total mean scores of FSFI and the subscales of sexual desire, arousal, orgasm, and satisfaction were significantly higher in the intervention group than in the control group after the intervention. In addition, postintervention pain mean scores in the intervention group were significantly lower than in the control group (*p* < 0.05).

**Conclusion** The results of the present study indicate that psychoeducational cognitive-behavioral counseling is effective in improving female sexual function. It is recommended to compare the effects of psychoeducational cognitive-behavioral counseling on sexual dysfunctions of couples and with a larger sample size in future research.

## Introduction

Sexual function, which is a part of the health of women, is a multidimensional phenomenon that is affected by many biological and psychological factors.^1^ ^2^ The World Health Organization (WHO) considers that sexual health “is a state of physical, emotional, mental and social wellbeing in relation to sexuality; it is not merely the absence of disease, dysfunction or infirmity.” This definition calls attention to the inter-related nature of the physical, mental and social dimensions of sexuality, and importantly, the notion of sexual well-being. Sexuality is a fundamental part of being human. “Sexual health” requires “a positive and respectful approach to sexuality and sexual relationships, as well as the possibility of having pleasurable and safe sexual experiences, free of coercion, discrimination and violence.” Sexual health as one's harmony of mind, feeling, and body that leads to the completion of personality, communication, and love.^3^

Therefore, any disorder that causes dissonance and, consequently, dissatisfaction with sex, can lead to sexual dysfunction.^4^

Sexual dysfunction is defined as a person's inability to have one or all stages of sexual activity including desire, arousal, and erection/ejaculation for men, vaginal moisture for women, and orgasm.^5^ Sexual dysfunction of women is one of the most common problems that ∼ between 40 and 45% of women suffer from.^6^ These dysfunctions are a chain of psychosexual disorders that manifest as disorders of sexual desire, arousal, orgasm, and pain during intercourse.^7^ These disorders have various origins such as anatomical, physiological, and psychological factors.^4^ ^8^

There are two general methods of pharmacological and nonpharmacological interventions to treat sexual disorders.^8^ ^9^ Nonpharmacological measures include cognitive-behavioral sex therapies, which are nowadays one of the most common approaches in the treatment of psychosexual and psychological disorders.^10^ ^11^ Cognitive-behavioral therapy (CBT) is a combination of cognitive and behavioral approaches that help the patient identify his distorted thinking patterns and inefficient behaviors.

Sex education is a process in which individuals acquire the necessary information and knowledge about sexuality and form their attitudes, beliefs, and values. This process contributes to healthy sexual development, interpersonal relationships, body image, gender roles and maintains the mental health of individuals in the community. Lack of information or misinformation about sexuality increase the risk of developing sexual disorders.^12^

A review study by ter Kuile et al,^8^ who examined CBTs in female sexual dysfunction, revealed that the best and most common therapeutic approach is the cognitive-behavioral approach. In their study on the effectiveness of cognitive-behavioral therapy in patients with vaginismus, ter Kuile et al^13^ showed that this type of treatment increased the frequency and reduced fear of sexual intercourse. In addition, in the studies by Salehzadeh et al^14^ and Hoyer et al,^15^ cognitive-behavioral training significantly decreased sexual disorders.

Sexuality and sexual behavior have become a taboo in Iran due to religious prohibitions. Therefore, addressing the issue of sexual need has always been associated with social shame. Concerning the attention to the training and counseling of sexuality, a literature review did not find interventional studies in Iran applying eclectic methods. The present study aimed to investigate the effectiveness of psychoeducational and cognitive-behavioral counseling on female sexual dysfunction in a little city in the southeast of Iran.

## Methods

### Study Design and Setting

The present study is a clinical trial with intervention and control groups in a general clinic of an educational hospital in Zarand, Iran. The study population consisted of women referring to the general clinic with various problems, in 2018.

### Sample Size

Initially, 31 women were estimated for each group (control and intervention groups). Regarding the probability of dropout, 35 subjects were considered in each group. The following formula was used to estimate the sample size.



Based on the previous study by Salehzadeh et al,^14^ sd1 and sd2 values were set at 0.4 for the standard deviation (SD) of the intervention group and 0.9 for the SD of the control group, respectively. A test power of 80%, a test error of 0.05, and an accuracy of 0.5 were considered.

### Sampling

The convenience sampling method was used. Subjects with inclusion criteria were included in the study and were divided into intervention and control groups in a quasi-random manner based on sampling days (odd and even). The inclusion criteria (to remove secondary sexual dysfunctions) included: 1) married, living with a spouse and aged > 25 years old; 2) having no disease affecting sexual function before the study (such as systemic diseases including liver failure, kidney failure, heart disease, diabetes, thyroid failure, malignant disease, and undesirable sexual experience), 3) not taking antihypertensive drugs, antidepressants, antihistamines, contraceptive pills, alcohol and drug abuse before the study; 4) not having any previous sexual disorders diagnosed in the women or their spouses by the specialists based on the self-report of the individual 5) not suffering from infertility; 6) obtaining a Female Sexual Function Index FSFI score lower than the cutoff point in the questionnaire^16^ and 7) not being in pregnancy, breastfeeding or menopause periods. The exclusion criteria for the present study included unwillingness to collaborate with the study as well as absence in more than one session.

### Instrument

The data collection tools in the present study include:

1- **Demographic questionnaire**

The demographic questionnaire included name, surname, age, education, number of children, marriage duration, method of contraception, and telephone number.

2- **Female sexual function index (FSFI)**

Rosen et al developed the 19-item FSFI. The questionnaire measures female sexual function in six independent domains, namely desire, arousal, lubrication, orgasm, satisfaction, and pain. The answers to the questions are based on the Likert five- and six-point scales. The number of items in the domains is not equal in the FSFI, so, the item scores in each domain were added and then multiplied by the factor number to make the domains equal in weight. The factor numbers for sexual desire, arousal, lubrication, orgasm, satisfaction, and pain are 0.6, 0.3, 03, 0.4, 0.4 and 0.4, respectively. The scores considered for the items are 1–5 and 0–5. A score of zero indicates that the person has not been sexually active for 4 weeks. The total scale score is obtained by adding the scores of the six domains together. Thus, the scoring is such that the higher the score, the better the sexual function. The maximum score for each domain will be 6, and it will be 36 for the whole scale to make the domains equal in weight. The minimum score for the sexual desire domain will be 1.2, it will be 0 for sexual arousal, lubrication, orgasm, and pain, it will be 0.8 for the satisfaction domain, and it will be 2 for the whole scale. The cut off point for the whole scale is 28.^18^

**Validity and reliability:** In the study by Rosen et al,^18^ the retest validity coefficient was reported properly for all six domains (r = 0.79 to 0.86) and the whole questionnaire (r = 0.88). The Cronbach α coefficient was in addition measured to be ≥ 0.82 for internal reliability. Divergent validity of this scale was obtained by the Locke-Wallace marital adjustment test for the whole scale (*p* ≤ 0.001, r = 0.41), indicating appropriate validity of this scale^18^. In the study of Wiegel et al,^19^ the Cronbach α coefficient for the internal reliability of the whole scale and all 6 domains was reported to be from good to excellent (> 9.0). The validity of the questionnaire showed a significant difference between the scores of the patient group and the control group in all 6 domains (*p* < 0.001). Mohammadi et al^17^ investigated the validity and reliability of this questionnaire in two groups of female sexual dysfunction and control. The reliability of the scale and subscales was obtained by calculating the Cronbach α coefficient, which was calculated > 0.70 for all individuals, indicating good reliability of this instrument.^17^

### Data Collection

The researchers entered the research setting (The clinic of Imam Ali Hospital) after obtaining the necessary permits from the University (Code of Ethics: IR.KMU.REC.1397.429) and enrolling the study in the Iranian Registry of Clinical Trials (IRCT) (IRCT20170611034452N6). Then the purpose of the study was explained to women with inclusion criteria referring to the research setting. After receiving verbal consent, pretest data were collected by a demographic questionnaire and the FSFI, which were completed in a self-fulfillment manner. After receiving the questionnaires, 70 women (8 of whom were considered as dropouts) who obtained score ≤ 28 in the FSFI were contacted and invited to participate in the study. Written consent was taken from the participants during a personal meeting. The objectives and methodology of the present study were in addition fully explained to the participants. The subjects were then randomly divided into intervention and control groups based on sampling days (even and odd). The intervention group was asked to participate in 8 counseling sessions (two/week/1.5 hour).^18^ ^19^ ^20^ The sessions were held in a 17-person group and in an 18-person group in the hospital hall.

The objectives of the training sessions during eight sessions of psychoeducational and CBT counseling were as follows:

**Session One:** importance of sex in married women, understanding the concept of sexual dysfunction and cognitive-behavioral counseling and its purpose, explaining the natural sex cycle, classifying sexual dysfunctions.

**Session two:** sexual desire disorders, factors affecting sexual desire in men and women, therapeutic interventions based on replacing cognitive errors in sexuality and training of behavioral techniques to improve sexual desire.

**Session three:** sexual arousal disorders, factors affecting sexual arousal of males and females, therapeutic interventions based on replacing cognitive errors in sexual arousal and training of behavioral techniques for reinforcement of arousal.

**Session four:** factors affecting lubrication in sex, therapeutic techniques to maintain and increase lubrication during sex.

**Session five:** Orgasm, affective factors and orgasmic disorders in men and women, therapeutic interventions based on replacing cognitive mental backgrounds which affect orgasm problems and training of behavioral techniques for reducing these problems such as sensate focused therapy, on-and-off techniques.

**Session six:** pain/penetration disorders in women (physical and nonphysical reasons) focusing on nonphysical cognitive issues, fear and anxiety and training of behavioral techniques such as Kegel exercises, systemic desensitization and gradual dilatation for vaginismus and dyspareunia.

**Session seven:** Factors affecting sexual satisfaction focusing in marital adjustment and intimacy, mindfulness on the pleasure of sex, behavioral techniques in afterplay.

**Session eight:** Discussing about women most common issues, summary of the previous sessions and conclusion.

The control group did not receive any intervention but they could apply for attending similar new counseling sessions after the present study. Then, posttest was performed in both groups after 1 month, and the results were statistically analyzed.

### Data Analysis

Data were analyzed by PASW Statistics for Windows, Version 18 (SPSS Inc., Chicago, IL, USA). Data have been reported based on frequency distribution tables (number and percent), central tendency and dispersion (mean and SD). Mean and SD were used to describe the score of sexual dysfunction. The Mann-Whitney test was used to determine the homogeneity of the two groups in terms of quantitative demographic variables due to the non-normality of the quantitative data. The chi-squared and Fisher exact tests were used to determine the homogeneity of the two groups in terms of qualitative variables. Parametric statistical tests (independent *t*-test to compare intervention and control groups, paired *t*-test to study the groups before and after intervention) were used according to the objectives of the study and parametric conditions (normal distribution and equality of variances).

### Ethical Considerations

The present study was approved by the Ethics Committee of the Kerman University of Medical Sciences with the Code of Ethics (IR.KMU.REC.1397.429), and it was registered in the IRCT (IRCT20170611034452N6). At the beginning of the sampling process, the study objectives were explained to the target population, and verbal consent was obtained. After completing the initial sampling, the aims and method of the study were fully explained to the participants who met the inclusion criteria and written consent was taken from them. In addition, the participants were assured that their information was kept confidential and anonymous and that the research results would be used and published only for research purposes and they could withdraw at any time.

## Results

Table 1 shows the demographic characteristics of the subjects. There was no significant difference between the two groups in demographic characteristics (*p* > 0.05) (Table 1).

**Table 1:** Demographic characteristics of subjects in two groups of intervention and control

Group Variable		Intervention		Control		*p-value*
		Frequency	Percent	Frequency	Percent	
Age (years old)	≤ 31	18	54.5	21	60.0	0.64
	> 31	15	45.5	14	40.0	
Education	School	7	21.2	9	25.7	0.33
	Diploma	13	39.4	18	51.4	
	Higher	13	39.4	8	22.9	
Children (number)	≤ 1	6	18.2	6	17.1	0.95
	2	14	42.4	14	40.0	
	≥ 3	13	39.4	15	42.9	
Marriage duration	≤ 5	14	42.4	18	51.4	0.45
	> 5	19	57.6	17	48.6	
Contraception method	None	4	12.1	6	17.1	0.84
	Condom	18	54.5	18	51.4	
	IUD	11	33.3	11	31.4	

Abbreviation: IUD, intrauterine device.

The sexual function score was not significantly different between the intervention and control groups before the intervention (*p* < 0.05). The total score of sexual function was significantly higher in the intervention group than in the control group after the intervention. In addition, the Mann-Whitney test showed that except for the lubrication domain, the scores of sexual desire, arousal, orgasm, and satisfaction were significantly higher in the intervention group than in the control group. In addition, the postintervention pain score in the intervention group was significantly lower than in the control group (*p* < 0.05) (Table 2).

**Table 2:** Comparison of female sexual function in the intervention and control groups before and after the intervention

	Before					After				
Variable	Intervention		Control		*p-value*	Intervention		Control		*p-value*
	Mean	SD	Mean	SD		Mean	SD	Mean	SD	
Sexual desire	6.03	2.31	6.17	2.51	0.79	7.61	1.32	6.00	2.10	0.001
Arousal	12.00	3.64	13.40	3.30	0.1	16.24	1.94	12.97	2.87	< 0.0001
Lubrication	11.03	1.88	11.54	1.69	0.25	11.09	1.18	11.40	1.29	0.38
Orgasm	9.61	2.09	9.26	1.75	0.46	10.18	1.01	9.40	1.22	<0.01
Satisfaction	9.45	2.32	9.69	2.19	0.52	12.76	1.06	9.94	2.25	< 0.0001
Pain	9.09	2.84	8.94	3.0	0.91	5.70	1.29	9.14	2.70	< 0.0001
Total	21.80	2.77	22.34	2.93	0.32	24.22	1.72	22.31	2.47	< 0.0001

Abbreviation: SD, standard deviation.

Z*= Mann-Whitney test; T*= independent-t-test.

There was a statistically significant increase in the mean scores of sexual desire, arousal, and satisfaction in the intervention group compared with before intervention as well as a significant reduction in the mean score of the pain domain. The mean score of lubrication did not change significantly after the intervention compared with before the intervention, and the increase in the mean score of the orgasm was not statistically significant. Furthermore, the total mean of sexual function scores was significant before and after the intervention. (*p* < 0.05) (Table 3).

**Table 3:** Comparison of female sexual function in the intervention group

Variable	Before intervention		After intervention		Test statistic	*p-value*
	Mean	SD	Mean	SD		
Sexual desire	6.03	2.31	7.61	1.32	Z*= - 4.23	< 0.0001
Arousal	12.00	3.64	16.24	1.94	Z= - 4.87	< 0.0001
Lubrication	11.03	1.88	11.09	1.18	Z= - 0.21	0.82
Orgasm	9.61	2.09	10.18	1.01	Z= - 1.95	0.051
Satisfaction	9.48	2.32	12.76	1.06	Z= - 4.8	< 0.0001
Pain	9.09	2.84	5.70	1.29	Z= - 4.87	< 0.0001
Total	21.80	2.77	24.22	1.72	T*= - 7.99	< 0.0001

Abbreviation: SD, standard deviation.

Z*= Wilcoxon test; T*= paired *t*-test.

There was no statistically significant decrease in the mean scores of sexual desire, arousal, and lubrication in the control group after the intervention compared with before the intervention (*p* > 0.05). An increase in the mean score of orgasm, satisfaction, and pain in the control group was not statistically significant after the intervention compared with before the intervention (*p* > 0.05). There was no significant difference in the total mean score of sexual function in the control group before and after the intervention (*p* > 0.05) (Table 4).

**Table 4:** Comparison of female sexual function in the control group

Variable	Before intervention		After intervention		Test statistic	*p-value*
	Mean	SD	Mean	SD		
Sexual desire	6.17	2.51	6.00	2.10	Z*= - 0.67	0.49
Arousal	13.40	3.30	12.97	2.87	Z= - 0.91	0.36
Lubrication	11.54	1.69	11.40	1.29	Z= - 0.91	0.35
Orgasm	9.26	1.75	9.40	1.22	Z= - 1.05	0.29
Satisfaction	9.69	2.19	9.94	2.25	Z= - 1.14	0.25
Pain	8.94	3.0	9.14	2.70	T*= - 0.85	0.4
Total	22.34	2.93	22.31	2.47	Z= 0.38	0.7

Abbreviation: SD, standard deviation.

Z*= Wilcoxon test; t*= paired-t-test.

## Discussion

The present study investigated the effectiveness of psychoeducational cognitive-behavioral counseling on female sexual function. The results of the present study suggest that this type of counseling is effective in improving female sexual function.

Ziaee et al^20^ studied the sexual dysfunction of married female students. Their results showed that the mean score of sexual function was lower in both control and intervention groups in all six components compared with the present study. The reasons for this difference can be at the different statistical population of the two studies as well as the duration of marriage because, in the study of Ziaee et al,^20^ the mean duration of marriage in the intervention and control groups was 29.4 and 22.4 months, respectively. However, in the present study, 48.6% of individuals in the control group and 57.6% of individuals in intervention group had been married for > 5 years.^20^ One possible reason may be lack of proper information and experience of young couples regarding marital sexual relationships.^20^ ^21^ ^22^ ^23^ ^24^ ^25^ ^26^ ^27^ ^28^ In addition, in the study of Ziaee et al,^20^ as well as in the study of Babakhani et al,^21^ the mean scores of all six domains of sexual desire, arousal, lubrication, orgasm, satisfaction and pain had a significant difference in the experimental group before and after the intervention. Furthermore, the mean total score of sexual function in the intervention group increased in two previous studies as well as in the present study after the intervention compared with before the intervention, and this difference was statistically significant.^20^ ^21^ In the study by ter Kuile et al,^8^, cognitive-behavioral counseling increased female sexual function in all six components and the total score of sexual function that was consistent with the results of the present study. In the study of Brotto et al,^22^ the mean total score of sexual function in the intervention group was 20.19 before the intervention, which reached 25.39 after the intervention. This difference was statistically significant, and this increase was in line with the results of the present study.^22^

Jalilian et al^23^ studied the effect of sexual skills training with the cognitive-behavioral method on sexual dysfunction in 40 infertile women aged between 22 and 36 years old. The results showed that the mean scores of sexual desire, arousal, satisfaction, orgasm, and lubrication significantly improved after three training sessions per week. However, pain score changes were not significant. The mean total score of sexual function in the intervention group increased from 21.80 to 24.22. This mean decreased in the control group.^23^ However, in the present study, the two domains of orgasm and lubrication did not improve significantly, but the improvement of the pain score was significant, which is inconsistent with the results of Jalilian et al.^23^ One of the reasons for this inconsistency in these two domains may be the duration of the intervention, which was 10 sessions (one session/week) in their study, longer than in the present study.

In addition, the results of the present study indicate that the mean scores of the six components of sexual desire, arousal, lubrication, orgasm, satisfaction, and pain were not significantly different in the control group before and after the intervention. However, in the study by Ziaee et al,^20^ the mean scores of sexual pain, satisfaction and orgasm decreased after the intervention. In the study of Ziaee et al.^20^ the mean score of sexual function decreased 2 months and 10 weeks after the intervention. However, re-evaluation and follow-up were performed after 1 month in the present study.^20^ ^23^

Although we provided a comprehensive explanation in the counseling and training package on the factors affecting lubrication and therapeutic interventions, counseling did not have a significant effect on the lubrication factor in the present study. Cultural factors, shyness, embarrassment, poor sexual function of the partner,^24^ and poor sex education of the partner^25^ are factors that may influence lubrication, and these factors might have influenced our ineffective consultation about lubrication. On the other hand, the results of the structured review by Weinberger et al^26^ showed that, despite significant improvement in certain areas of sexual dysfunction in women, neither drug therapy nor psychotherapy interventions lead to complete improvement of the disease. Furthermore, some studies did not support the effect of sex consultation on female sexual function with female genital mutilation.^27^

There were in addition limitations to the present study because of the relatively small number of individuals in each group. Therefore, the results of the present study are less generalizable to the female community. Studies with more subjects, time framework, and financial opportunities are required. In addition, the researchers had some difficulty in justifying people to participate and continue the research at the sampling and data gathering stages because of the taboo of sexuality in our society.

## Conclusion

The results indicated that the psychoeducational cognitive-behavioral counseling had a significant positive effect on increasing female sexual function. These results may be usable for healthcare institutions to improve sexual function and satisfaction in women. It is recommended to compare the effects of psychoeducational cognitive-behavioral counseling on sexual dysfunction of couples and to use more subjects in future researches.
